# Improved Root Growth by Liming Aluminum-Sensitive Rice Cultivar or Cultivating an Aluminum-Tolerant One Does Not Enhance Fertilizer Nitrogen Recovery Efficiency in an Acid Paddy Soil

**DOI:** 10.3390/plants9060765

**Published:** 2020-06-19

**Authors:** Hao Qing Zhang, Xue Qiang Zhao, Yi Ling Chen, Jia Lin Wang, Ren Fang Shen

**Affiliations:** 1 State Key Laboratory of Soil and Sustainable Agriculture, Institute of Soil Science, Chinese Academy of Sciences, Nanjing 210008, China; hqzhang@issas.ac.cn (H.Q.Z.); lynne.chen@amway.com (Y.L.C.); jlwang@issas.ac.cn (J.L.W.); rfshen@issas.ac.cn (R.F.S.); 2 Institute of Soil Science, University of Chinese Academy of Sciences, Beijing 100049, China

**Keywords:** aluminum toxicity, rice, lime, fertilizer nitrogen recovery efficiency, nitrogen uptake, root

## Abstract

The root is the main site of nitrogen (N) acquisition and aluminum (Al) toxicity. The objective of this study is to investigate whether liming and cultivation of an Al-tolerant rice (*Oryza sativa* L.) cultivar can improve root growth, thereby increasing N acquisition by rice plants in acid paddy soil. Two rice cultivars (‘B690’, Al-sensitive, and ‘Yugeng5’, Al-tolerant) were cultivated with ^15^N-labeled urea, and with or without lime in an acid paddy soil (pH 4.9) in pots. We examined root and shoot growth, soil pH, soil exchangeable Al, N uptake, ^15^N distribution in plant-soil system, and fertilizer N recovery efficiency. Results showed that liming improved the root growth of ‘B690’ by decreasing soil exchangeable Al concentrations, in both N-limited and N-fertilized soils. Liming enhanced the N uptake of ‘B690’ only in the absence of N fertilizer. The root weight of ‘Yugeng5’ was greater than that of ‘B690’ without lime, but the two cultivars showed similar N uptake. The fertilizer N recovery efficiency and N loss did not differ significantly between limed and non-limed conditions, or between the two rice cultivars. Thus, liming an Al-sensitive rice cultivar and cultivating an Al-tolerant one improves root growth, but does not enhance fertilizer N recovery efficiency in the present acid paddy soil.

## 1. Introduction

Acid soils (defined as pH < 5.5) are present in more than 50% of the cultivated and cultivable land area worldwide [1]. Therefore, the cultivation of crops in acid soils is important for agricultural production and food security. However, agricultural productivity in acid soils is limited by a combination of soil stress factors, including the high contents of available aluminum (Al), manganese (Mn), iron (Fe) and protons (H^+^), and deficiencies of phosphorus (P), nitrogen (N), calcium (Ca) and magnesium (Mg) [1,2]. Aluminum toxicity is generally considered as the primary limiting factor for plant growth in acid soils [3,4]. Aluminum, as the most abundant metal element in the Earth’s crust, is generally insoluble in neutral to slightly acid soils [5]. When soil pH is lower than 5.0, toxic Al ions are released into the soil solution, where they inhibit root growth and ultimately reduce crop yields [3,4]. The main symptom of Al toxicity is the inhibition of root growth [6,7]. Roots are the main organs through which plants acquire various mineral nutrients from the soil [8,9]. The inhibitory effects of Al on root growth may result in reduced nutrient uptake from acid soils [10], which will further aggravate nutrient deficiencies in acid soils. The improvement of crop productivity in acid soils depends on increased tolerance to Al toxicity and increased nutrient uptake [10].

Nitrogen is an essential macro-element, that plays a key role in plant growth and development. Nitrogen deficiency is a major limiting factor for crop production in acid soils [11]. Large amounts of N fertilizers are applied to meet the growing food demands of the increasing population [12]. At the same time, excessive and irrational use of N fertilizers results in negative environmental effects, such as water pollution and greenhouse gas emissions [12]. Nitrogen fertilizer in ammonium form is a major driver of soil acidification, through soil nitrification and plant ammonium uptake [13,14]. Soil acidification caused by ammonium-based N fertilizer increases toxic Al concentrations in soil [15]. Because the N uptake capability of roots is poor under Al toxicity, more N fertilizer is required to meet the N requirements of crops, in order to maintain a high yield. This can lead to further soil acidification and aggravated Al toxicity. This vicious circle can seriously restrict crop production in acid soils. Under the extreme conditions of soil acidification induced by long-term N fertilization, no crop grain can be obtained [16,17]. Therefore, the study of interactions between N fertilization and Al toxicity is an important issue for agricultural production in acid soils.

Rice (*Oryza sativa* L.) is the staple food for nearly 50% of the world’s population. Approximately 13% of rice produced worldwide is grown in acid soils [1]. Rice crops account for about 10% of the total N fertilizer applied worldwide [18]. The fertilizer N recovery efficiency (FNRE) of rice plants is around 30% [19,20]. More than 60% of the N fertilizer applied to rice crops is lost through leaching, denitrification, volatilization and runoff [18]. Moreover, the overuse of N fertilizer has resulted in significant acidification of paddy soils in recent years [13]. The average soil pH in paddy fields in South China decreased by 0.59 from 1988 to 2013, mainly because of N fertilization [21]. Therefore, there is an urgent need to improve the FNRE of rice and reduce paddy soil acidification.

Although there have been numerous studies investigating the interactions between Al and N in rice [10,22,23], the effects of Al-alleviating strategies on the FNRE of rice are rarely studied. In various agricultural practices, liming is a traditional and effective way to alleviate Al toxicity through increasing soil pH [24]. Different rice cultivars differ widely in their Al tolerance [7,23]. The inhibitory effect of Al on N uptake by plants may depend on that plant’s level of Al tolerance [10]. For example, Al was found to inhibit N uptake by wheat root tips in an Al-sensitive genotype, but to stimulate it in an Al-tolerant genotype [25]. Thus, liming and the cultivation of Al-tolerant rice cultivars may alleviate Al toxicity and improve N uptake in acid soils. However, there is still little experimental evidence concerning whether these strategies can increase the FNRE of rice plants grown in acid paddy soils. In this context, the objective of this study is to investigate the effect of liming on rice growth, grain yield, and the fertilizer N fate and recovery efficiency of ^15^N-labeled urea, for Al-tolerant and Al-sensitive rice cultivars in acid paddy soil.

## 2. Results

### 2.1. Comparison of Al Tolerance between Two Rice Cultivars

**Figure 1:**
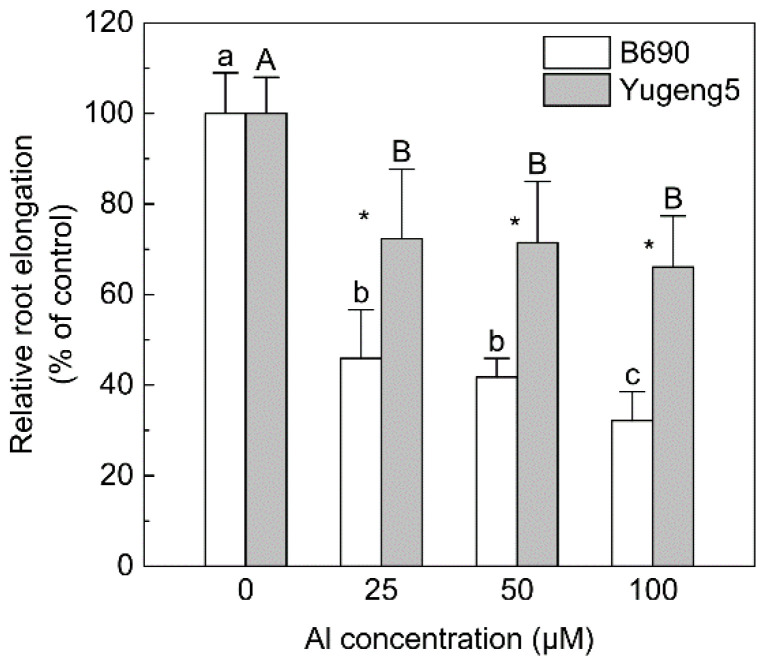
Comparison of aluminum (Al) tolerance between two rice cultivars, ‘B690’ and ‘Yugeng5’. Two-day-old rice seedlings were exposed to 0.5 mM CaCl_2_ solution (pH 4.9) containing 0, 25, 50 or 100 μM Al for 24 h. Relative root elongation (%) is the ratio of root elongation with Al to root elongation without Al. Different lowercase and capital letters above bars indicate significant differences among different Al concentrations for the same rice cultivar (*p* < 0.05, Duncan’s multiple range test). Asterisks indicate significant differences between the two rice cultivars at the same Al concentration (*p* < 0.05, independent-sample *t* test).

Since the two rice subspecies *indica* and *japonica* often show significant differences in Al tolerance [23,26], two rice cultivars, ‘B690’ (*O. sativa* subsp. *indica*) and ‘Yugeng5’ (*O. sativa* subsp. *japonica*), were used in this study. The Al tolerance of the two cultivars was tested in a hydroponic experiment. Aluminum decreased the relative root elongation of both rice cultivars compared with 0 μM Al. The relative root elongation of ‘Yugeng5’ was significantly higher than that of ‘B690’, at 25, 50 and 100 μM Al (Figure 1), indicating that ‘Yugeng5’ is relatively Al-tolerant, while ‘B690’ is Al-sensitive.

### 2.2. Effects of Lime and N on Biomass of Two Rice Cultivars

**Figure 2:**
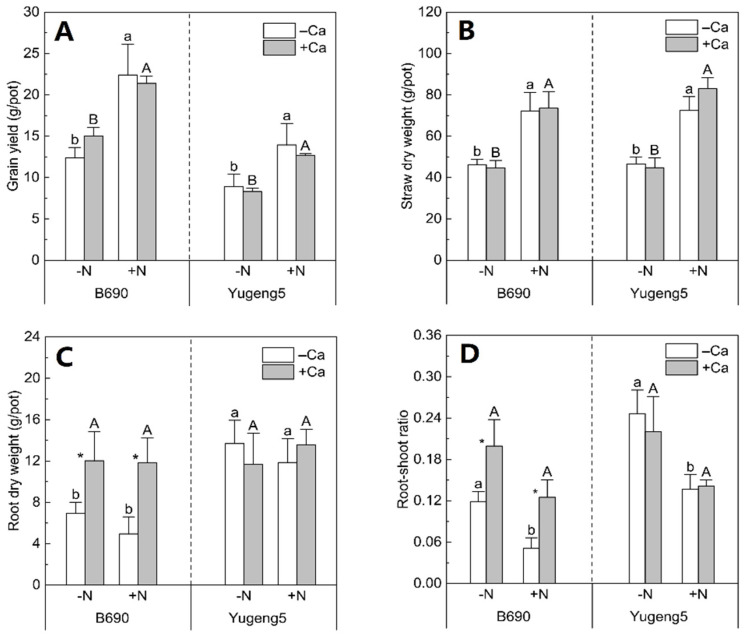
Effects of nitrogen (N) and lime on biomass of two rice cultivars, ‘B690’ and ‘Yugeng5’. (**A**), Grain yield; (**B**), straw dry weight; (**C**), root dry weight; (**D**), root–shoot ratio. Root–shoot ratio; ratio of root dry weight to aboveground dry weight. Two rice cultivars were grown in pots in a greenhouse without (−N) or with (+N) N fertilizer, in the absence (−Ca) or presence (+Ca) of lime, till maturity. Different lowercase and capital letters above bars indicate significant differences between −N and +N treatments under −Ca and +Ca conditions for the same rice cultivar, respectively (*p* < 0.05, independent-sample *t* test). Asterisks indicate significant differences between −Ca and +Ca treatments for the same rice cultivar (*p* < 0.05, independent-samples *t* test).

Nitrogen fertilizer increased the grain yield and straw dry weight of both rice cultivars by 42–85% (Figure 2A,B), but did not affect their root dry weight (Figure 2C). Consequently, N fertilizer decreased the root/shoot ratio of both rice cultivars by 36–57% (Figure 2D). Compared with the −Ca (absence of lime) treatment, +Ca (presence of lime) did not affect the grain yield or straw dry weight of either rice cultivar (Figure 2A,B). Liming did not affect the root dry weight and root/shoot ratio of Al-tolerant rice cultivar ‘Yugeng5’, but significantly increased those parameters in the Al-sensitive rice cultivar ‘B690’ by 68–143% (Figure 2C,D). The root dry weight of ‘Yugeng5’ was about two times that of ‘B690’ in −Ca (absence of lime) conditions, but the root dry weight of the two cultivars was similar in +Ca (presence of lime) conditions (Figure 2C). These results show that lime improved the root growth of Al-sensitive rice cultivar ‘B690’, and that the Al-tolerant rice cultivar ‘Yugeng5’ formed roots bigger than those of the Al-sensitive rice cultivar ‘B690’ in the absence of lime.

### 2.3. Effects of Lime and N on N Uptake of Two Rice Cultivars

**Figure 3:**
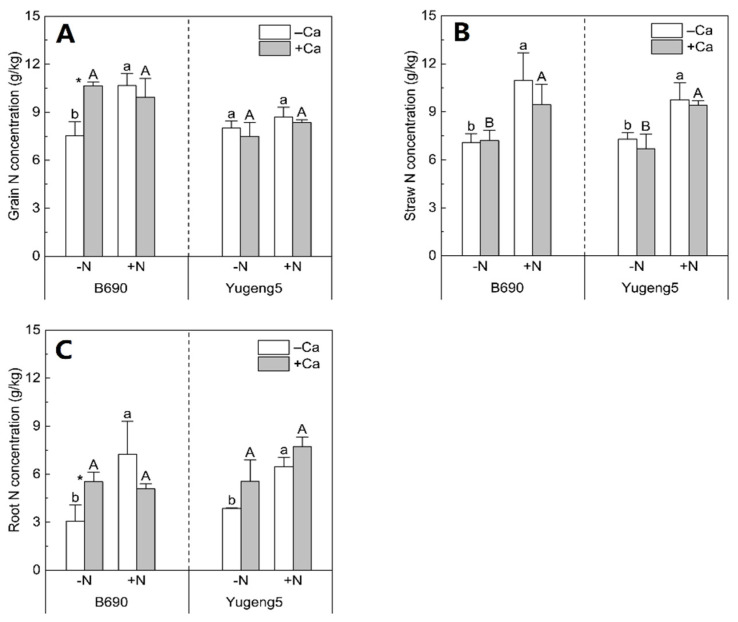
Effects of nitrogen (N) and lime on N concentrations in rice cultivars ‘B690’ and ‘Yugeng5’. (**A**), Grain N concentration; (**B**), straw N concentration; (**C**), root N concentration. Two rice cultivars were grown in pots in a greenhouse without (−N) or with (+N) N fertilizer, in the absence (−Ca) or presence (+Ca) of lime, till maturity. Different lowercase and capital letters above bars indicate significant differences between −N and +N treatments under −Ca and +Ca conditions for the same rice cultivar, respectively (*p* < 0.05, independent-sample *t* test). Asterisks indicate significant differences between −Ca and +Ca treatments for the same rice cultivar (*p* < 0.05, independent-sample *t* test).

Nitrogen fertilizer increased the grain N concentration of only ‘B690’, by 42%, without lime (Figure 3A), the straw N concentration of both cultivars by 31–55%, with and without lime (Figure 3B), and the root N concentration by 68–136% in both cultivars, in the absence of lime (Figure 3C). Lime increased the N concentrations in grains and roots by 41% and 80%, respectively, only in ‘B690’ under −N conditions (Figure 3A,C). However, liming did not significantly affect the N concentrations in grains, straws or roots under other conditions (Figure 3). Therefore, lime and N fertilizer had much greater effects on the N concentration in the Al-sensitive cultivar ‘B690’ than on that in the Al-tolerant cultivar ‘Yugeng5’.

**Figure 4:**
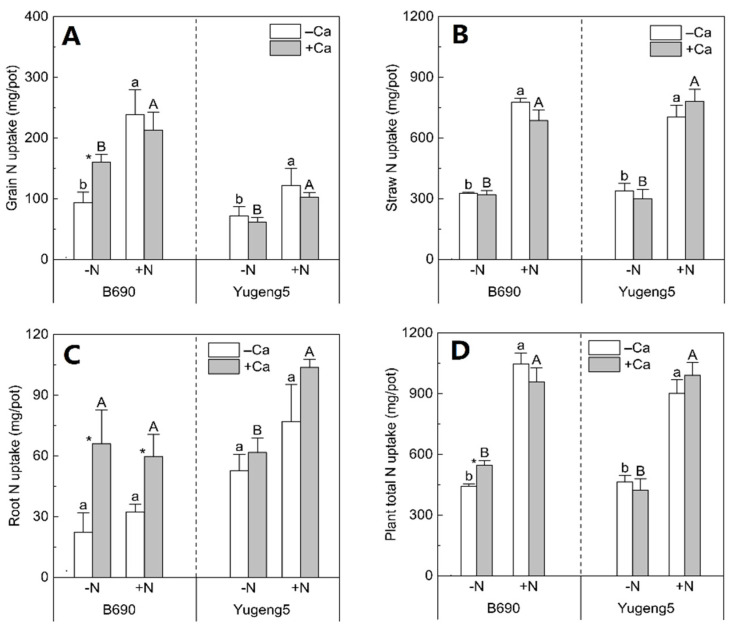
Effects of nitrogen (N) and lime on N uptake of rice cultivars ‘B690’ and ‘Yugeng5’. (**A**), Grain N uptake; (**B**), straw N uptake; (**C**), root N uptake; (**D**), plant total N uptake. Two rice cultivars were grown in pots in a greenhouse without (−N) or with (+N) N fertilizer, in the absence (−Ca) or presence (+Ca) of lime, till maturity. Different lowercase and capital letters above bars indicate significant differences between −N and +N treatments under −Ca and +Ca conditions for the same rice cultivar, respectively (*p* < 0.05, independent-sample *t* test). Asterisks indicate significant differences between −Ca and +Ca treatments for the same rice cultivar (*p* < 0.05, independent-samples *t* test).

Compared with −N treatments, N fertilizer treatments increased the N uptake of grains and straws, and the total N uptake of the two cultivars, by 33–161% in the presence and absence of lime (Figure 4A,B,D). The N uptake of grains, straws and roots of ‘Yugeng5’ did not differ significantly between −Ca (absence of lime) and +Ca (presence of lime) treatments, regardless of N fertilization (Figure 4A–C). However, compared with the −Ca (absence of lime) treatment, +Ca (presence of lime) increased the root N uptake of ‘B690’ by 195% and 85%, under −N and +N conditions, respectively (Figure 4C); +Ca (presence of lime) increased the grain N uptake and total N uptake of ‘B690’, by 71% and 24%, respectively, under −N conditions (Figure 4A,D). These results indicate that lime had more positive effects on the N uptake of the Al-sensitive cultivar ‘B690’ than on that of Al-tolerant cultivar ‘Yugeng5’, especially under N-limited conditions.

### 2.4. Effects of Lime and N on Soil pH and Exchangeable Al

**Figure 5:**
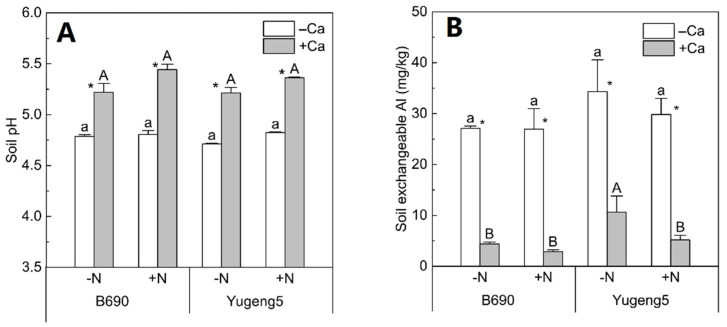
Soil pH and exchangeable Al after harvesting of two rice cultivars ‘B690’ and ‘Yugeng5’. (**A**), Soil pH; (**B**), exchangeable Al. Two rice cultivars were grown in pots in a greenhouse without (−N) or with (+N) N fertilizer in the absence (−Ca) or presence (+Ca) of lime till maturity. Different lowercase and capital letters above bars indicate significant differences among different N treatments under −Ca and +Ca conditions, respectively (*p* < 0.05, Duncan’s multiple range test). Asterisks indicate significant differences between −Ca and +Ca treatments (*p* < 0.05, independent-sample *t* test).

The soil pH after rice harvest was significantly improved, by 0.53, by liming in all treatments, while there was no significant difference in soil pH between −N and +N, or between the two rice cultivars (Figure 5A). In contrast, liming greatly decreased the soil exchangeable Al, by about 81%, for the two rice cultivars (Figure 5B).

### 2.5. Fate of ^15^N-Labeled Fertilizer N in Soil–Plant Systems

**Figure 6:**
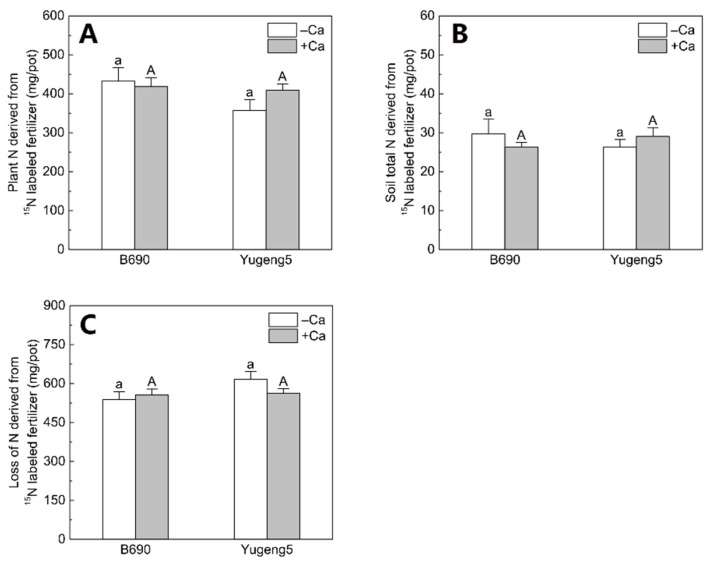
Pathway of ^15^N-labeled fertilizer nitrogen (N) in plant–soil systems after the harvesting of two rice cultivars ‘B690’ and ‘Yugeng5’. Two rice cultivars were grown in pots in a greenhouse without (−N) or with (+N) N fertilizer, in the absence (−Ca) or presence (+Ca) of lime, till maturity. (**A**), Plant N derived from ^15^N-labeled fertilizer; (**B**), soil total N derived from ^15^N-labeled fertilizer; (**C**), loss of N derived from ^15^N-labeled fertilizer. Loss of fertilizer was calculated as the difference between amount of applied fertilizer N and the sum of fertilizer N recovered in plant and soil. Different lowercase and capital letters above bars indicate significant differences between the two rice cultivars under –Ca and +Ca conditions, respectively (*p* < 0.05, independent-sample *t* test). Independent-sample *t* test showed no significant difference between −Ca and +Ca treatments for the two rice cultivars at 5% level.

**Figure 7:**
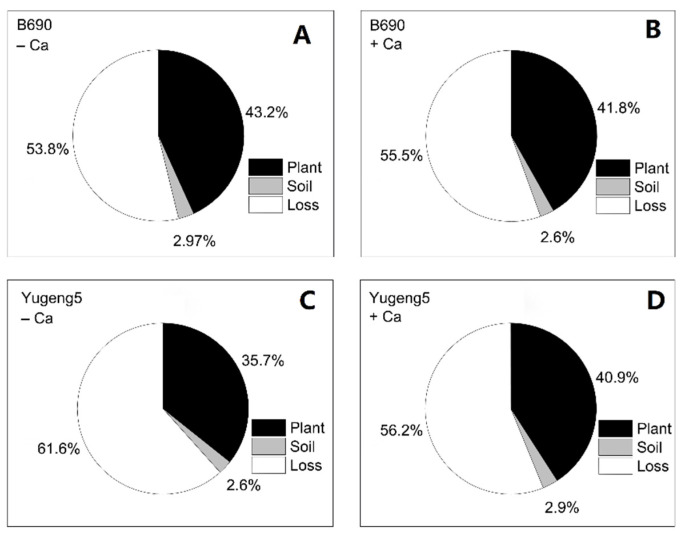
Distribution of ^15^N-labeled fertilizer nitrogen (N) in plant-soil system after harvesting of two rice cultivars ‘B690’ and ‘Yugeng5’. (**A**), B690, −Ca; (**B**), B690, +Ca; (**C**), Yugeng5, −Ca; (**D**), Yugeng5, +Ca. Two rice cultivars were grown in pots in a greenhouse without (−N) or with (+N) N fertilizer, in the absence (−Ca) or presence (+Ca) of lime, till maturity.

There were no significant differences in plant N, soil N or lost N derived from ^15^N-labeled fertilizer, between the −Ca (absence of lime) and +Ca (presence of lime) treatments, or between the two rice cultivars (Figure 6). Of the applied fertilizer N, 35.7–43.2% was taken up by rice plants, 2.6–2.97% remained in soils, while 53.8–61.6% was lost to the environment (Figure 7).

### 2.6. Fertilizer N Recovery Efficiency

**Figure 8:**
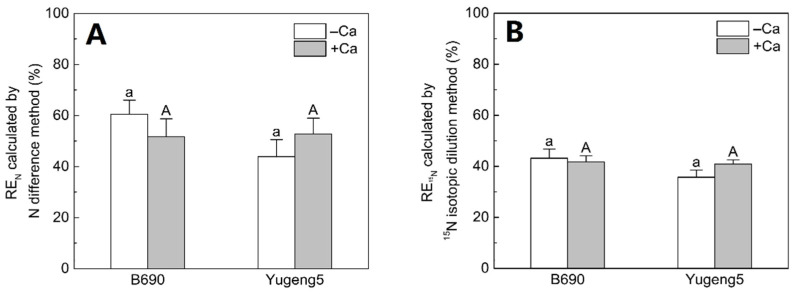
Effect of liming on fertilizer nitrogen (N) recovery efficiency (FNRE) of the two rice cultivars ‘B690’ and ‘Yugeng5’. (**A**), FNRE calculated by N difference method; (**B**), FNRE calculated by ^15^N isotopic dilution method. Two rice cultivars were grown in pots in a greenhouse without (−N) or with (+N) N fertilizer, in the absence (−Ca) or presence (+Ca) of lime, till maturity. Different lowercase and capital letters above bars indicate significant differences between the two rice cultivars under −Ca and +Ca conditions, respectively (*p* < 0.05, independent-sample *t* test). Independent-sample *t*-test showed no significant difference between −Ca and +Ca treatments for the two rice cultivars at 5% level.

The FNRE, as calculated using the N difference method and the ^15^N isotope dilution method, did not differ significantly between the −Ca (absence of lime) and +Ca (presence of lime) treatments, or between the two rice cultivars (Figure 8). These results show that liming and cultivation of an Al-tolerant rice cultivar did not improve FNRE in the present acid paddy soil.

## 3. Discussion

### 3.1. Liming Improved Growth of Roots but Not Aboveground Parts of an Al-Sensitive Rice Cultivar

*O. sativa* subsp. *indica* cultivars are generally Al-sensitive, while *O. sativa* subsp. *japonica* cultivars are Al-tolerant [23,26]. Consistent with this, the *indica* cultivar ‘B690’ was found to be Al-sensitive, while the *japonica* cultivar ‘Yugeng5’ was Al-tolerant in the hydroponic experiment in the present study. The main criteria for estimating phytotoxicity of Al in acid soils is the soil’s exchangeable Al content [27]. The results in this study indicate that liming increased soil pH and decreased soil exchangeable Al concentrations, which would alleviate Al toxicity to rice. In the present study, liming improved the root growth of ‘B690’ but not ‘Yugeng5’, indicating that Al toxicity inhibited the root growth of ‘B690’, but not ‘Yugeng5’, under these soil conditions. Thus, the results of both the hydroponic and soil culture experiments demonstrate that the *indica* rice cultivar ‘B690’ is Al-sensitive, while the *japonica* cultivar ‘Yugeng5’ is Al-tolerant.

Roots play an important role in the acquisition of nutrients and water, and are closely associated with the growth and development of the aboveground parts of plants [28,29]. Previous studies have detected a positive linear relationship between the root weight and grain yield of rice [30,31]. However, the results of the present study show that liming enhanced the root growth, but not the straw weight and grain yield, of the Al-sensitive rice cultivar ‘B690’. The results of the previous study also indicate that Al toxicity decreases the dry weight of roots, but not that of shoots [22], indicating that shoot growth is not completely dependent on roots. Similarly, Chang and Sung [32] found that liming increased the dry weight of rice roots, but slightly decreased the dry weight of the aboveground parts by 0.7%. A negative correlation between maize yield and root number was observed, because of the competition between roots and aboveground parts for N [33]. As root establishment and maintenance are fueled by assimilates produced by the aboveground parts, a larger root system could result in greater energy consumption, and therefore be unfavorable for grain yield formation [29]. Thus, larger root biomass does not guarantee larger aboveground biomass.

In contrast with the results of the present study, several other studies reported that liming can increase the grain yields and straw dry weights of rice plants under certain conditions [34,35,36]. For example, Fageria and Knupp [34] reported the beneficial effects of liming on the grain yield of upland rice under dryland conditions. As water deficiency is a limiting factor for the growth of upland rice, liming may improve root growth, and therefore increase the water uptake of upland rice, leading to increased grain yield. Ai et al. [35] observed that liming increased the grain yield of rice in acid sulfate soil (pH 4.07), and this pH is much lower than the pH 4.90 of the soil used in the present study. Liming may be much more effective in increasing the grain yield of rice plants growing in strongly acid soils. Liao et al. [36] found that liming interacted with straw retention to increase rice grain yield, probably because of increased organic matter decomposition and enhanced N mineralization. Thus, it may be possible for liming to improve the aboveground growth of rice under certain conditions, such as in upland areas, in strongly acid soils, and in soils with high organic material inputs.

### 3.2. Liming Enhanced N Uptake of Al-Sensitive Rice Cultivar in the Absence of N Fertilizer

In a review by Adams and Martin [37], liming was suggested to improve N uptake and use through restoring a favorable soil pH environment for plant growth. In the present study, liming improved the N uptake of Al-sensitive rice cultivar ‘B690’ in the absence of N fertilizer. Liming can enhance the N uptake of rice growing in soil with low N availability in two ways: by increasing soil available N and/or by enhancing N acquisition by roots. Several reports have indicated that liming can increase the soil’s available N concentrations and plant N uptake, by enhancing soil N mineralization [36,38,39,40]. In the present study, liming did not improve the N uptake of Al-tolerant rice cultivar ‘Yugeng5’ in the absence of N fertilizer, suggesting that liming might not enhance soil N mineralization under these conditions. Thus, the liming-enhanced N uptake of ‘B690’ in the absence of N fertilizer was not related to an increase in soil N mineralization by liming. Liming increased the root biomass of ‘B690’, which enhanced its ability to take up N in the absence of N fertilizer. As liming did not increase the root biomass of the Al-tolerant cultivar ‘Yugeng5’, it did not enhance the N uptake of this cultivar in the absence of N fertilizer. Therefore, the liming-increased N uptake of Al-sensitive rice cultivar ‘B690’, in the absence of N fertilization, may be associated with increased root biomass, rather than enhanced soil N mineralization.

In the presence of N fertilizer, liming increased the root biomass of the Al-sensitive rice cultivar ‘B690’, but did not enhance its N uptake. Previous studies have shown that roots function in enhancing N acquisition mainly when N is limited [41,42], and that root distribution is more critical for plant N uptake under N-limited conditions [43]. Some studies reported that efficient N uptake under N-deficient conditions was dependent on a larger root system [44,45]. However, another study found that the amount of N uptake by maize under N-sufficient conditions was determined by the shoot growth potential, rather than the root size [46]. This may explain why the increased root biomass after the application of lime did not result in higher N uptake in the Al-sensitive rice cultivar, ‘B690’, in the presence of N fertilizer.

Although several studies have reported that liming increases plant N uptake in acid soils [36,38,39,40], fewer studies have paid attention to the effects of liming on the FNRE. Weligama et al. [47] found that the effects of liming on the FNRE of ^15^N-labeled fertilizers in wheat depended on the forms of N in acid soil (pH 3.6): liming decreased the FNRE of urea, increased that of ammonium, and had no effects on that of nitrate. In the present study, liming improved the root growth of the Al-sensitive rice cultivar, but it did not increase the FNRE of urea. This is mainly because liming did not increase rice grain yield, straw weight or N uptake in the presence of N fertilizer.

### 3.3. Cultivation of an Al-Tolerant Rice Cultivar Did Not Improve FNRE

Detailed knowledge of Al-N interactions in the soil–plant system may reveal strategies to synergistically enhance plant Al tolerance and N efficiency [10]. In a quantitative trait locus genetic (QTL) analysis, the genomic region regulating the seminal root length’s response to N was co-located with the main QTL region linked to Al tolerance in rice [48]. The cultivation of Al-tolerant rice cultivars may be an alternative approach to improving FNRE in acid paddy soils. In the present study, the root dry weight of the Al-tolerant rice cultivar ‘Yugeng5’ was greater than that of the Al-sensitive rice cultivar ‘B690’ in the absence of lime. However, the bigger root system of the Al-tolerant rice cultivar did not lead to a higher FNRE than that of the Al-sensitive rice cultivar in the present study. A recent report also indicated that the N use efficiency of *indica* rice cultivars is generally superior to that of *japonica* cultivars in paddy fields [49]. Therefore, it is necessary to breed rice cultivars that are both Al-tolerant and N-efficient in acid paddy soils.

Roots play an important role in the acquisition of N from soils [8,9]. In the present study, we detected a negative correlation between root/shoot ratio and straw N concentration, indicating that a larger root system does not necessarily result in higher straw N uptake (Figure S1), especially under conditions of high soil N availability, as discussed above. In addition, there was no correlation between root biomass and rice N uptake (Figure S2). Together, these results indicate that a larger root system does not necessarily result in higher N uptake or FNRE of rice plants.

Plants can increase nutrient acquisition from soils via roots, either by enlarging the root system or by increasing the nutrient transport rate across the root membrane [46]. A larger root system allows plants to contact more nutrients by occupying a larger soil volume, while a higher nutrient transmembrane rate helps plants to absorb more nutrients in a shorter time [46]. In dryland areas, N mobility in soils might be limited by water shortage, and root size may become more important for dryland plants in acquiring more N from soils. Craine et al. [50] observed a strong relationship between the fine root biomass and the depletion of soil N for 11 grassland plants. Tian et al. [51] found that the greater N acquisition ability of an N-efficient maize cultivar was attributed to the coordination of leaf and root growth, but not to the root N uptake rate. However, under flooded conditions, differences in the uptake of nutrients, including N, among different rice cultivars were not attributed to variations in root morphology, but to the differences in ion uptake characteristics [52]. When N is highly soluble and movable in the soils, root length and density are not limiting factors for N acquisition [53]. Therefore, the larger root system, due to the cultivation of an Al-tolerant rice cultivar and liming, did not lead to more efficient N uptake or use in the presence of N fertilizer in the present study. The higher fertilizer N uptake and recovery efficiency of rice plants in acid paddy soil may not depend on a larger root system, but is probably related to other characteristics, such as the efficiency of the NH_4_^+^ and NO_3_^−^ transport systems.

## 4. Materials and Methods

### 4.1. Hydroponic Experiment

Two rice cultivars, ‘B690’ (*Oryza sativa* subsp. *indica*) and ‘Yugeng5’ (*O. sativa* subsp. *japonica*), were used in this study. The Al tolerance of the two cultivars was tested in a hydroponic experiment. Seeds were soaked for 24 h in deionized water, and then germinated for 24 h on moist filter paper at 33 °C in the dark. Subsequently, the germinated seeds were placed on a framed net floating on 0.5 mM CaCl_2_ solution (pH 4.9), and grown in a growth chamber at 22 °C. This pH was selected according to the pH of the soil used in the subsequent soil culture experiment. After 2 days of growth in the dark, the root length of seedlings was 2–3 cm, and 12 uniform seedlings, which were considered as 12 replicates, were selected from each cultivar for Al treatment. The selected seedlings were treated with 0, 25, 50 or 100 μM Al (AlCl_3_·6H_2_O) in 0.5 mM CaCl_2_ solutions (pH 4.9) in pots (1 L pot^−1^). After growing in a growth chamber at 22 °C in the dark for 24 h, the root length of each seedling was measured with a ruler before and after the Al treatments. Since the inhibition of root elongation is the most direct and obvious symptom of Al toxicity, relative root elongation is widely used to evaluate the Al tolerance of different plants [7,23]. Relative root elongation (%) was calculated as the ratio of root elongation with Al treatment to root elongation without Al treatment. This index was used to evaluate the Al tolerance of the two cultivars.

### 4.2. Pot Experiment

A pot experiment was conducted in a greenhouse at the Institute of Soil Science, Chinese Academy of Sciences (32°03′ N, 118°47′ E). The soil used for the soil culture experiment was taken from a paddy field at Yingtan Red Soil Ecological Experiment Station (28°12′ N, 116°55′ E), Jiangxi Province, China. This soil is derived from Quaternary Red Clay, and is classified as Plinthudults in accordance with the US system of Soil Taxonomy. The collected soil was air-dried, sieved (<2 mm), and mixed well before use. The basic properties of the experimental soil are summarized in Table 1.

The pot experiment included three factors, lime (+Ca) or not (−Ca), N fertilizer (+N) or not (−N), and two rice cultivars (‘B690’ and ‘Yugeng5’), as described above. Each treatment had three replicates. Five kilograms air-dried soil was weighed into each pot (depth 25 cm; internal diameter 16 cm). Lime was applied at 1 g CaCO_3_/kg soil (equivalent to 1.3 ton/ha in the field). Nitrogen fertilizer was applied as ^15^N-labeled urea (20.16 atom % excess) at 200 mg N/kg soil (equivalent to 260 kg/ha in the field). The ^15^N-labeled fertilizer was produced by the Shanghai Research Institute of Chemical Industry, Shanghai, China. Phosphorus (P) and potassium (K) were applied to all pots as monopotassium phosphate (KH_2_PO_4_) and potassium chloride (KCl) at 50 mg P/kg soil (equivalent to 65 kg/ha in the field) and 150 mg K/kg soil (equivalent to 195 kg/ha in the field), respectively. The application amounts of fertilizers and lime were designed according to the local farmer traditional fertilization. All fertilizers were mixed well with soil prior to planting rice.

The rice seeds were germinated as described above, and the seedlings were first grown in N-free modified Kimura B macronutrient and Arnon micronutrient nutrient solution, as described previously [22]. After 16 days of growth, uniform seedlings were selected and transplanted into soils (four seedlings/pot). The experimental pots were randomly arranged. During the culture experiment, the temperature in the greenhouse was in the range of 28–34 °C. Deionized water was added to the soil every 2 days to maintain flooded conditions. When the rice plants were mature, the grains, straws and roots were separated, washed with deionized water, killed by heating at 120 °C for 1 h, and then oven-dried at 75 °C to a constant weight. The oven-dried plant materials were weighed and then ground to pass through a 0.149 mm sieve for analysis of N concentrations and ^15^N abundance.

### 4.3. Analytical Methods

Soil pH was measured with a pH meter (PB-21, Sartorius, Göttingen, Germany) in a 1:2.5 soil:water mixture. Soil exchangeable Al was extracted with 1 M KCl, and measured by inductively coupled plasma–atomic emission spectroscopy (ICP-AES) (ICAP 6300, Thermo Fisher, Waltham, MA, USA). Total N in soil was measured by a CNS elemental analyzer (VarioMAX, Elementar, Hanau, Germany). Soil ammonium (NH_4_^+^-N) and nitrate (NO_3_^−^-N) were extracted with 2 M KCl, and their concentrations were measured with a flow analyzer (San^++^ System, Skalar Analytical B.V., Breda, Netherlands). Soil available P was determined by the Bray extraction method, and K by flame photometry (FP640, Shanghai Precision & Scientific Instrument Inc., Shanghai, China). Soil organic matter (SOM) was determined using a colorimetric method after oxidizing organic matter in soil samples with K_2_Cr_2_O_7_ in concentrated sulfuric acid. For plant materials, the N concentration was measured using the indophenol blue colorimetry method after digestion with sulfuric acid and 30% hydrogen peroxide [54]. The abundance of ^15^N in the plants and soils was measured by elemental analyzer-isotopic ratio mass spectrometry (EA-IRMS) (Flash-2000 Delta V Advantage, Thermo Fisher, Waltham, MA, USA).

### 4.4. Calculation Methods for Fertilizer N Loss and FNRE

The ^15^N fertilizer-derived N recovery in rice plants and soils was computed according to Zhang et al. [55]. Loss of N derived from ^15^N-labeled fertilizer was the difference between the total fertilizer N applied and the sum of the ^15^N fertilizer-derived N recovery in rice plants and the soil N pool. The FNRE was calculated by the N difference method (RE_N_) and the ^15^N isotope dilution method (RE_15N_) [56]. The N difference method requires a zero-N fertilizer control that is not required for the ^15^N isotope dilution method.

The following calculation was used to determine fertilizer N recovery efficiency using the N difference method (RE_N_, %): RE_N_ = (total plant N uptake in the N fertilized treatments – total plant N uptake in the unfertilized treatments)/total N applied × 100

The calculation of fertilizer N recovery efficiency using the ^15^N isotope dilution method (RE_15N_, %) was as follows: RE_15N_ = fertilizer-derived N recovery in rice plant/total N applied × 100

### 4.5. Statistical Analyses

All statistical analyses were performed with IBM SPSS Statistics for Windows Version 19.0 (IBM Corporation, Armonk, NY, USA). Significant differences in relative root elongation among four Al concentrations were assessed using one-way ANOVA followed by Duncan’s multiple range test at the 5% level. The effects of liming practice (L), N fertilization (N), different cultivars (C) and their interactive effect on each index were evaluated by a two-way ANOVA (Table S1). Furthermore, the significance of differences in various indices between ‘B690’ and ‘Yugeng5’, between −Ca (absence of lime) and +Ca (presence of lime) treatments, and between −N and +N treatments were assessed by an independent-samples *t* test at the 5% level. All graphs were drawn using Origin Pro 2015 (OriginLab, Northampton, MA, USA). In all graphs, data are shown as mean ± standard deviation (SD).

## 5. Conclusions

Liming improved the root growth of the Al-sensitive rice cultivar ‘B690’, but did not improve the root growth of the Al-tolerant rice cultivar ‘Yugeng5’ or the aboveground growth of either cultivar. Liming enhanced the N uptake of ‘B690’ in the absence of N fertilizer, but not in the presence of N fertilizer, whereas liming did not enhance the N uptake of ‘Yugeng5’ in the presence or absence of N fertilizer. There were no significant differences in the FNRE and N loss between limed and non-limed conditions. Although the Al-tolerant rice cultivar ‘Yugeng5’ had a larger root system than that of the Al-sensitive rice cultivar ‘B690’ in the absence of lime, the FNRE of ‘Yugeng5’ was not higher than that of ‘B690’. We conclude that, although liming an Al-sensitive rice cultivar and the cultivation of an Al-tolerant rice cultivar helped rice plants to form a larger root system, these measures did not improve FNRE in the present acid paddy soil. The effects of liming and cultivating Al-tolerant rice cultivar on fertilizer N recovery efficiency should be further examined in the field.

## Figures and Tables

**Table 1:** Initial properties of soil used in the pot and field experiments.

pH	TN (mg/g)	SOM (mg/g)	NO_3_^−^-N (mg/kg)	NH_4_^+^-N (mg/kg)	Available P (mg/kg)	Available K (mg/kg)
4.9	1.2	11.3	2.4	4.4	29.3	94.1

TN, total nitrogen; SOM, soil organic matter; NO_3_^−^-N, nitrate-nitrogen; NH_4_^+^-N, ammonium-nitrogen; available P, available phosphorus; available K, available potassium.

## Supplementary Materials

The following are available online at https://www.mdpi.com/2223-7747/9/6/765/s1, Table S1: Analysis of variances of the effects of liming practice (L), N fertilization (N), different rice cultivars (C) and their interactions (*P* value), Figure S1: Correlation analyses of root–shoot ratio with nitrogen (N) concentration in rice, Figure S2: Correlation analyses of root dry weight with N uptake in rice.

## References

[1] Von Uexküll H.R., Mutert E. (1995). Global extent, development and economic impact of acid soils. Plant Soil.

[2] Zhao X.Q., Chen R.F., Shen R.F. (2014). Coadaptation of plants to multiple stresses in acidic soils. Soil Sci..

[3] Kochian L.V., Piñeros M.A., Hoekenga O.A. (2005). The physiology, genetics and molecular biology of plant aluminum resistance and toxicity. Plant Soil.

[4] Ma J.F., Chen Z.C., Shen R.F. (2014). Molecular mechanisms of Al tolerance in gramineous plants. Plant Soil.

[5] Kochian L.V. (1995). Cellular mechanisms of aluminum toxicity and resistance in plants. Annu. Rev. Plant Physiol. Plant Mol. Biol..

[6] Ryan P.R., Ditomaso J.M., Kochian L.V. (1993). Aluminum toxicity in roots: An investigation of spatial sensitivity and the role of the root cap. J. Exp. Bot..

[7] Ma J.F., Shen R., Zhao Z., Wissuwa M., Takeuchi Y., Ebitani T., Yano M. (2002). Response of rice to Al stress and identification of quantitative trait loci for Al tolerance. Plant Cell Physiol..

[8] Garnett T., Conn V., Kaiser B.N. (2009). Root based approaches to improving nitrogen use efficiency in plants. Plant Cell Environ..

[9] Li X., Zeng R., Liao H. (2016). Improving crop nutrient efficiency through root architecture modifications. J. Integr. Plant Biol..

[10] Zhao X.Q., Shen R.F. (2018). Aluminum–nitrogen interactions in the soil–plant system. Front. Plant Sci..

[11] Fageria N.K., Baligar V.C. (2001). Improving nutrient use efficiency of annual crops in Brazilian acid soils for sustainable crop production. Commun. Soil Sci. Plant Anal..

[12] Zhu Z.L., Chen D.L. (2002). Nitrogen fertilizer use in China—Contributions to food production, impacts on the environment and best management strategies. Nutr. Cycl. Agroecosys..

[13] Guo J.H., Liu X.J., Zhang Y., Shen J.L., Han W.X., Zhang W.F., Christie P., Goulding K.W., Vitousek P.M., Zhang F.S. (2010). Significant acidification in major Chinese croplands. Science.

[14] Liang L.Z., Zhao X.Q., Yi X.Y., Chen Z.C., Dong X.Y., Chen R.F., Shen R.F. (2013). Excessive application of nitrogen and phosphorus fertilizers induces soil acidification and phosphorus enrichment during vegetable production in Yangtze River Delta, China. Soil Use Manag..

[15] Schroder J.L., Zhang H., Girma K., Raun W.R., Penn C.J., Payton M.E. (2011). Soil acidification from long-term use of nitrogen fertilizers on winter wheat. Soil Sci. Soc. Am. J..

[16] Wang C., Zheng M., Song W., Wen S., Wang B., Zhu C., Shen R. (2017). Impact of 25 years of inorganic fertilization on diazotrophic abundance and community structure in an acidic soil in southern China. Soil Biol. Biochem..

[17] Afreh D., Zhang J., Guan D., Liu K., Song Z., Zheng C., Deng A., Feng X., Zhang X., Wu Y. (2018). Long-term fertilization on nitrogen use efficiency and greenhouse gas emissions in a double maize cropping system in subtropical China. Soil Tillage Res..

[18] Ghosh B.C., Bhat R. (1998). Environmental hazards of nitrogen loading in wetland rice fields. Environ. Pollut..

[19] Fageria N.K., Baligar V.C. (2003). Methodology for evaluation of lowland rice genotypes for nitrogen use efficiency. J. Plant Nutr..

[20] Peng S., Buresh R.J., Huang J., Zhong X., Zou Y., Yang J., Wang G., Liu Y., Hu R., Tang Q. (2010). Improving nitrogen fertilization in rice by site-specific N management. A review. Agron. Sustain. Dev..

[21] Zhou X., Zhou S., Xu M., Gilles C. (2015). Evolution characteristics and influence factors of acidification in paddy soil of southern China. Sci. Agric. Sin..

[22] Zhao X.Q., Shen R.F., Sun Q.B. (2009). Ammonium under solution culture alleviates aluminum toxicity in rice and reduces aluminum accumulation in roots compared with nitrate. Plant Soil.

[23] Zhao X.Q., Guo S.W., Shinmachi F., Sunairi M., Noguchi A., Hasegawa I., Shen R.F. (2013). Aluminium tolerance in rice is antagonistic with nitrate preference and synergistic with ammonium preference. Ann. Bot..

[24] Goulding K.W.T. (2016). Soil acidification and the importance of liming agricultural soils with particular reference to the United Kingdom. Soil Use Manag..

[25] Ikeda M., Yamanishi T. (1999). Accumulation of nitrogen supplied as ammonium in the root tips of aluminum-stressed wheat cultivars differing in aluminum sensitivity. J. Fac. Agric. Kyushu Univ..

[26] Famoso A.N., Clark R.T., Shaff J.E., Craft E., McCouch S.R., Kochian L.V. (2010). Development of a novel aluminum tolerance phenotyping platform used for comparisons of cereal aluminum tolerance and investigations into rice aluminum tolerance mechanisms. Plant Physiol..

[27] Hoyt P.B., Nyborg M. (1971). Toxic metals in acid soil: I. Estimation of plant-available aluminum. Soil Sci. Soc. Am. J..

[28] Lynch J. (1995). Root architecture and plant productivity. Plant Physiol..

[29] Yang J., Zhang H., Zhang J. (2012). Root morphology and physiology in relation to the yield formation of rice. J. Integr. Agric..

[30] Yamazaki K. (1989). Root system formation and its relation to grain yield in rice plants. Korean J. Crop Sci..

[31] Yamazki K., Harada J. (1982). The root system formation and its possible bearings on grain yield in rice plants. Jpn. Agric. Res. Q..

[32] Chang C.S., Sung J.M. (2004). Nutrient uptake and yield responses of peanuts and rice to lime and fused magnesium phosphate in an acid soil. Field Crop Res..

[33] Gallais A., Coque M. (2005). Genetic variation and selection for nitrogen use efficiency in maize: A synthesis. Maydica.

[34] Fageria N.K., Knupp A.M. (2014). Influence of lime and gypsum on growth and yield of upland rice and changes in soil chemical properties. J. Plant Nutr..

[35] Ai C., Liang G., Sun J., He P., Tang S., Yang S., Zhou W., Wang X. (2015). The alleviation of acid soil stress in rice by inorganic or organic ameliorants is associated with changes in soil enzyme activity and microbial community composition. Biol. Fertil. Soils..

[36] Liao P., Huang S., van Gestel N.C., Zeng Y., Wu Z., van Groenigen K.J. (2018). Liming and straw retention interact to increase nitrogen uptake and grain yield in a double rice-cropping system. Field Crop Res..

[37] Adams F., Martin J.B., Hauck R.D. (1984). Liming effects on nitrogen use and efficiency. Nitrogen in Crop Production.

[38] Nyborg M., Hoyt P.B. (1978). Effects of soil acidity and liming on mineralization of soil nitrogen. Can. J. Soil Sci..

[39] Bailey J.S. (1995). Liming and nitrogen efficiency: Some effects of increased calcium supply and increased soil pH on nitrogen recovery by perennial ryegrass. Commun. Soil. Sci. Plant Anal..

[40] Stevens R.J., Laughlin R.J. (1996). Effects of lime and nitrogen fertilizer on two sward types over a 10-year period. J. Agric. Sci..

[41] Liu J., Chen F., Olokhnuud C., Glass A.D.M., Tong Y., Zhang F., Mi G. (2009). Root size and nitrogen-uptake activity in two maize (*Zea mays*) inbred lines differing in nitrogen-use efficiency. J. Plant Nutr. Soil Sci..

[42] Kiba T., Krapp A. (2016). Plant nitrogen acquisition under low availability: Regulation of uptake and root architecture. Plant Cell Physiol..

[43] Richardson A.E., Barea J.M., McNeill A.M., Prigent-Combaret C. (2009). Acquisition of phosphorus and nitrogen in the rhizosphere and plant growth promotion by microorganisms. Plant Soil..

[44] Robinson D., Rorison I.H. (1983). Relationship between root morphology and nitrogen availability in a recent theoretical model describing nitrogen uptake from soil. Plant Cell Environ..

[45] Wang Y., Mi G., Chen F., Zhang J., Zhang F. (2004). Response of root morphology to nitrate supply and its contribution to nitrogen accumulation in maize. J. Plant Nutr..

[46] Peng Y., Niu J., Peng Z., Zhang F., Li C. (2010). Shoot growth potential drives N uptake in maize plants and correlates with root growth in the soil. Field Crop Res..

[47] Weligama C., Tang C., Sale P.W.G., Conyers M.K., Liu D.L. (2010). Application of nitrogen in NO_3_^−^ form increases rhizosphere alkalisation in the subsurface soil layers in an acid soil. Plant Soil..

[48] Ogawa S., Selvaraj M.G., Fernando A.J., Lorieux M., Ishitani M., McCouch S., Arbelaez J.D. (2014). N- and P-mediated seminal root elongation response in rice seedlings. Plant Soil..

[49] Zhang J., Liu Y.X., Zhang N., Hu B., Jin T., Xu H., Qin Y., Yan P., Zhang X., Guo X. (2019). *NRT1.1B* is associated with root microbiota composition and nitrogen use in field-grown rice. Nat. Biotechnol..

[50] Craine J.M., Wedin D.A., Chapin III F.S., Reich P.B. (2002). Relationship between the structure of root systems and resource use for 11 North American grassland plants. Plant Ecol..

[51] Tian Q., Chen F., Zhang F., Mi G. (2006). Genotypic difference in nitrogen acquisition ability in maize plants is related to the coordination of leaf and root growth. J. Plant Nutr..

[52] Teo Y.H., Beyrouty C.A., Norman R.J., Gbur E.E. (1995). Nutrient uptake relationship to root characteristics of rice. Plant Soil..

[53] Sinclair T.R., Vadez V. (2002). Physiological traits for crop yield improvement in low N and P environments. Plant Soil..

[54] Cataldo D.A., Schrader L.E., Youngs V.L. (1974). Analysis by digestion and colorimetric assay of total nitrogen in plant tissues high in nitrate. Crop Sci..

[55] Zhang H.Q., Zhao X.Q., Chen Y.L., Zhang L.Y., Shen R.F. (2019). Case of a stronger capability of maize seedlings to use ammonium being responsible for the higher ^15^N recovery efficiency of ammonium compared with nitrate. Plant Soil..

[56] Krupnik T.J., Six J., Ladha J.K., Paine M.J., van Kessel C., Mosier A.R., Syers K.J., Freney J.R. (2004). An assessment of fertilizer nitrogen recovery efficiency by grain crops. Agriculture and the Nitrogen Cycle.

