# Supplementary material for: Improved Root Growth by Liming Aluminum-Sensitive Rice Cultivar or Cultivating an Aluminum-Tolerant One Does Not Enhance Fertilizer Nitrogen Recovery Efficiency in an Acid Paddy Soil
Source: Plants (Basel). 2020 Jun 19;9(6):765. doi: 10.3390/plants9060765 (PMC7355884; doi:10.3390/plants9060765)
Supplement: Supplementary file 1 [file plants-09-00765-s001.pdf]

Supplementary Material

Table S1 Analysis of variances of the effects of liming practice (L), N fertilization (N), different rice cultivars (C) and their interactions (*P* value)

Sources of variation	Dry weight				N concentration			N uptake			Pathway of ^{15}N labeled fertilizer			Soil pH	Soil exchangeable Al	RE_N	RE_{15N}
	G	S	R	R/S ratio	G	S	R	G	S	R	Plant	Soil	Loss				
L	0.951	0.374	**0.015**	**0.035**	0.286	0.276	0.114	0.744	0.409	**<0.001**	0.363	0.838	0.355	**<0.001**	**<0.001**	0.987	0.363
N	**<0.001**	**<0.001**	0.618	**<0.001**	**0.013**	**<0.001**	**0.001**	**<0.001**	**<0.001**	**0.006**	-	-	-	**0.005**	0.079	-	-
C	**<0.001**	0.294	**0.003**	**0.001**	**<0.001**	0.471	0.184	**<0.001**	0.807	**<0.001**	0.070	0.842	0.062	0.355	**0.009**	0.116	0.070
L×N	0.240	0.136	0.212	0.694	**0.019**	0.514	**0.020**	**0.049**	0.614	0.948	-	-	-	0.126	0.714	-	-
C×L	0.337	0.355	**0.012**	**0.008**	**0.034**	0.823	0.193	0.165	0.064	0.125	0.137	0.090	0.101	0.844	0.815	0.083	0.137
C×N	0.070	0.341	0.602	0.433	0.540	0.663	0.597	**0.040**	0.656	**0.012**	-	-	-	0.913	0.203	-	-
L×N×C	0.40	0.337	0.661	0.515	**0.012**	0.374	0.05	0.082	**0.009**	0.137	-	-	-	0.294	0.954	-	-

G, grain; S, shoot; R, root; R/S ratio, root weight/shoot weight ratio; RE_N, the FNRE calculated using N difference method; RE_{15N}, the FNRE ^{15}N isotope dilution method. Values in bold indicate significant differences ($P < 0.05$).

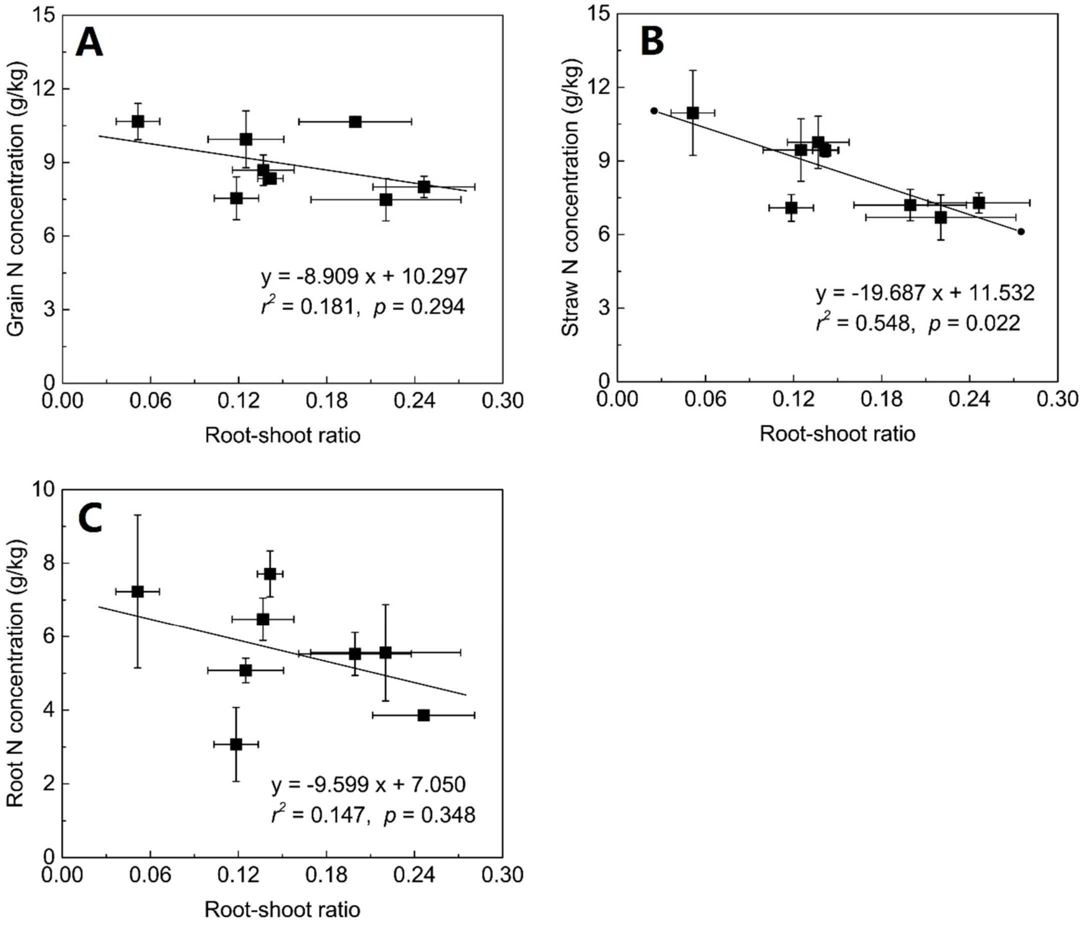

Figure S1. Correlation analyses of root-shoot ratio with nitrogen (N) concentration in rice. **(A)**, Grain N concentration; **(B)**, straw N concentration; **(C)**, root N concentration. Root-shoot ratio; ratio of root dry weight to aboveground dry weight. Two rice cultivars 'B690' and 'Yugeng5' were grown in pots in a greenhouse without (−N) or with (+N) N fertilizer in the absence (−Ca) or presence (+Ca) of lime till maturity.

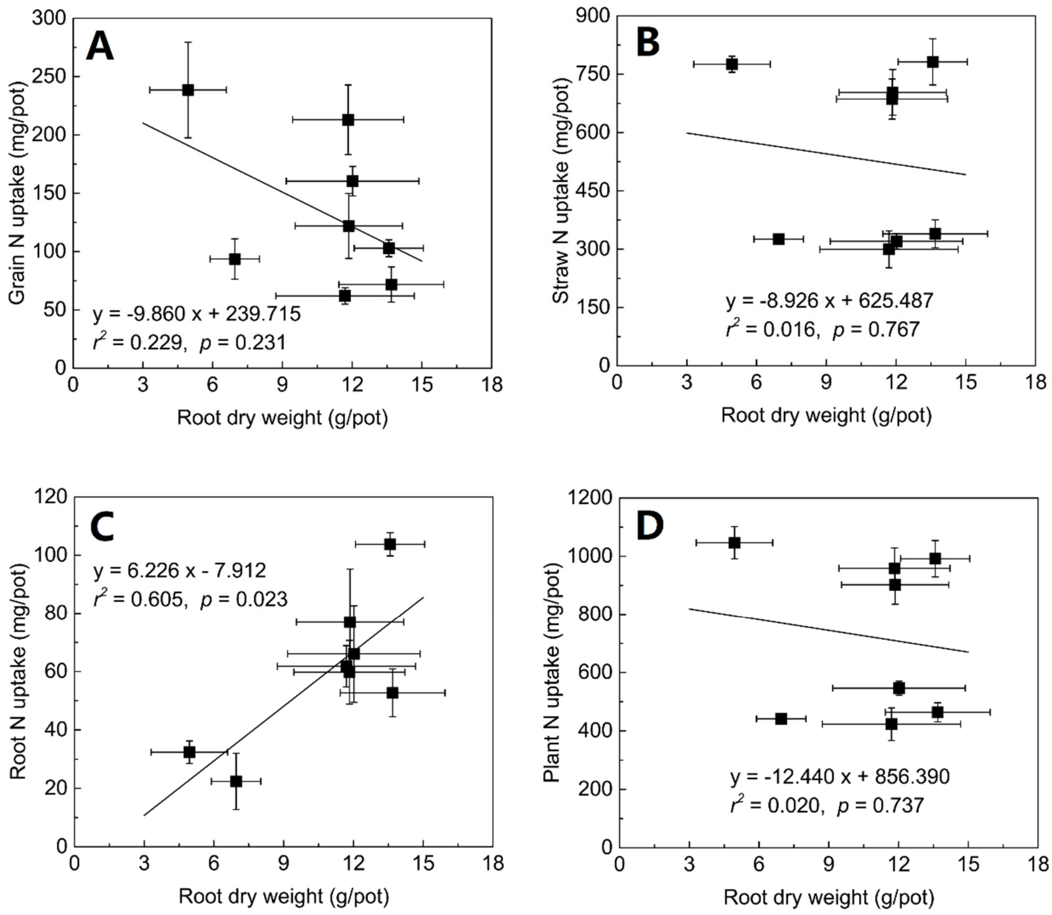

Figure S2. Correlation analyses of root dry weight with N uptake in rice. **(A)**, Grain N uptake; **(B)**, straw N uptake; **(C)**, root N uptake; **(D)** plant total N uptake. Two rice cultivars were grown in pots in a greenhouse without (−N) or with (+N) N fertilizer in the absence (−Ca) or presence (+Ca) of lime till maturity.
